# Pediatric Inflammatory Multisystem Syndrome Temporally Associated with SARS-CoV-2 Treated with Tocilizumab

**DOI:** 10.3390/pediatric12030029

**Published:** 2020-12-04

**Authors:** Carmen Niño-Taravilla, Yazmín P. Espinosa-Vielma, Hugo Otaola-Arca, Cecilia Poli-Harlowe, Lorena I. Tapia, Paula Ortiz-Fritz

**Affiliations:** 1 Pediatric Intensive Care Unit, Hospital Roberto del Río, Santiago 13108, Chile; pauortiz@hotmail.com; 2 Pediatric Intensive Care Unit, Clínica Indisa, Santiago 7500000, Chile; 3 Department of Pediatric Immunology and Rheumatology, Hospital Roberto del Río, Santiago 13108, Chile; y.espinosa.v@gmail.com (Y.P.E.-V.); cecipolih@gmail.com (C.P.-H.); 4 Department of Urology, Clínica Alemana, Santiago 13132, Chile; hugotaolarca@hotmail.com; 5 Clínica Alemana, Universidad del Desarrollo, Santiago 13132, Chile; 6 Department of Pediatric Infectiology, Hospital Roberto del Río, Santiago 13108, Chile; lorenaisabeltapia@gmail.com; 7 Virology Program, Institute of Biomedical Sciences, School of Medicine, Universidad de Chile, Santiago 13108, Chile

**Keywords:** SARS-COV-2, PIMS-TS, MIS-C, Tocilizumab

## Abstract

We describe a case of Pediatric Inflammatory Multisystem Syndrome temporally associated with SARS-CoV-2 (PIMS-TS) in an 8-year-old child. The patient developed multiorgan dysfunction, including mixed shock, cardiac dysfunction with myocarditis, pneumonia, acute kidney failure, and gastrointestinal involvement characterized by inflammation of the wall of the bowel and pancreatitis. After treatment with Tocilizumab and corticoid therapy, he presented clinical improvement and normalization of inflammatory markers. PIMS-TS is a new disease developed in a small percentage of patients, so a high degree of suspicion is necessary to establish the diagnosis. Supportive care is of paramount importance. The use of Tocilizumab to control the inflammatory response is likely to be beneficial, but the best immunotherapeutic agent has not yet been established. Randomized clinical studies should be run to determine the best treatment.

## 1. Introduction

COVID-19 in adults has been described to develop in three phases: early infection, pulmonary phase, and hyperinflammatory phase [1]. The latter phase has been associated with a cytokine storm [2,3] and secondary hemophagocytic lymphohistiocytosis [4], resulting in an ineffective cytotoxic response with elevated interleukin (IL)-6, which can suppress the normal activation of T lymphocytes.

The severe presentation of COVID-19 in children is infrequent. In Europe and the United States, there has been reported an increase in pediatric cases of Kawasaki disease (KD) and multisystem inflammatory syndrome probably related to the SARS-CoV-2 outbreak. In this context, the Royal College of Pediatrics and Child Health (RCPCH) proposed the definition of Pediatric Inflammatory Multisystem Syndrome temporally associated with SARS-CoV-2 (PIMS-TS) [5], also known as Multisystem Inflammatory Syndrome in Children (MIS-C) [6]. This syndrome includes persistent fever, inflammation, and evidence of single or multiorgan dysfunction (shock, cardiac, respiratory, renal, gastrointestinal, or neurological disorder), after the exclusion of any other microbial cause, but with evidence of COVID-19 infection (PCR SARS-CoV-2, serology and/or epidemiological link) [5,6].

Recent publications described case reports and case series with PIMS-TS or with Kawasaki-like disease; many of them were characterized by shock, requiring fluid resuscitation, and inotropic support, which in some series such as Whittaker et al. reached 50% [7,8,9,10]. In most cases, there was an epidemiological link with SARS-CoV-2, with a positive PCR swab and/or positive IgG, or family history of SARS-CoV-2 infection. 

The underlying immune mechanisms of this severe clinical presentation are unknown. Characterization of the infectious process and immune response during active viral replication and the possible post-infection immune-mediated mechanisms is being investigated [11,12]. Immunomodulatory therapy, including intravenous immunoglobulin (IVIG), corticosteroids, and biological drugs, has been used to curb the cytokine storm, together with intensive supportive care [5,6].

We report the first case of an 8-year-old patient with no comorbidities diagnosed with PIMS-TS treated with Tocilizumab in Chile.

## 2. Case Report

**Figure 1:**
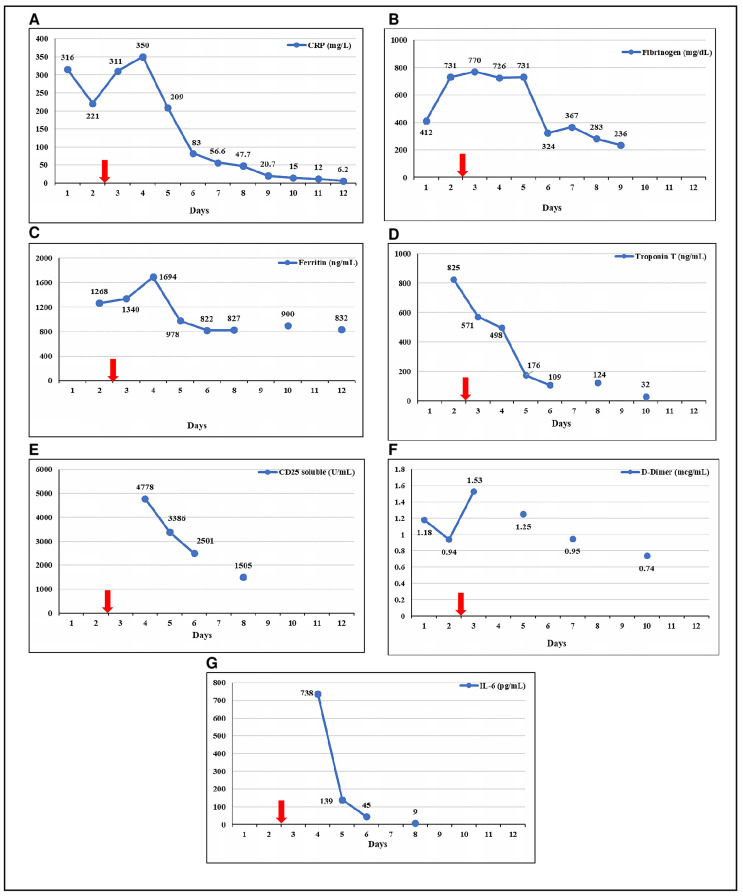
Evolution of inflammatory and cardiac parameters. (**A**) C reactive protein (CRP); (**B**) Fibrinogen; (**C**) Ferritin; (**D**) Troponin T; (**E**) CD25 soluble; (**F**) D-Dimer; (**G**) IL-6. Red arrow indicates the day of the administration of Tocilizumab. The last ferritin was taken on day 17 (200 ng/mL).

On 11 May 2020, an 8-year-old and previously healthy boy presented with a 6-day history of high fever, diarrhea, vomiting, and abdominal pain. His parents had had a positive nasopharyngeal swab PCR for SARS-CoV-2 a month before. On admission, he was febrile up to 39 °C, pale, tachycardic, and with predominantly diastolic hypotension. He presented diffuse abdominal pain without respiratory signs or symptoms. Blood, stool, and urine samples were obtained for cultures, and laboratory tests were performed. Empiric antibiotic therapy (cefotaxime) and fluid resuscitation with 20 mL/kg of saline solution were started. Blood tests showed metabolic acidosis, hyponatremia, slight increase in creatinine (up to 0.87 mg/dL), lactate (8 mg/dL), and a hypoalbuminemia (2 g/dL). Complete blood count was normal, including 10,000/mm^3^ leukocytes and 5300/mm^3^ lymphocytes. Inflammatory markers were elevated (Figure 1). Chest X-ray, electrocardiogram and echocardiogram were normal upon admission to the emergency department, whereas abdominal ultrasound revealed inflammation in the sigmoid colon. Due to maintained tachycardia and hypotension despite volume resuscitation, the patient was admitted to the pediatric intensive care unit (PICU). Nasopharyngeal swab PCR for SARS-CoV-2 resulted positive.

Upon admission to the PICU, a second ultrasound was performed, about 2 h after the previous one. It showed a mild left ventricular systolic dysfunction (50% ejection fraction) with normal coronary arteries. Given these findings, the study was expanded, and a Troponin T elevation was detected, with high total creatine kinase (CK) and normal CK-MB. The electrocardiogram was repeated, and it revealed a flattened ST segment in precordial leads. The patient developed a mixed shock (cardiogenic and distributive) requiring vasoactive support and invasive mechanical ventilation. On the second day of admission, a respiratory worsening was observed with increased oxygen requirements due to left basal pneumonia. Multisystem inflammatory syndrome associated with SARS-CoV-2 was suspected since all bacterial cultures were negative and inflammatory parameters continued to increase (Figure 1) along with an evident clinical worsening. In this context, a single dose of Tocilizumab (8 mg/kg) was administered on the second day of PICU admission. Methylprednisolone (1 mg/kg/day) and antithrombotic prophylaxis (enoxaparin, 1 mg/kg/day) were also initiated. No antiviral agents were prescribed. Serum IL-6 levels could not be tested before the use of Tocilizumab, but serial determinations were obtained the following days.

The patient’s condition progressively improved in the following 48 h, with subsequent normalization of cardiac function and laboratory results (Figure 1). Vasoactive support was discontinued the following day after the administration of Tocilizumab, and the patient was extubated one day later. A moderate increase in pancreatic enzymes was detected on day 4, with a normal abdominal ultrasound. Corticosteroid therapy (prednisone, 1 mg/kg/day) was continued until ferritin levels were under 500 mg/dL, and enoxaparin prophylaxis was maintained for ten days. Antibiotic treatment was suspended after 5 days, with the result of negative blood cultures. Serial echocardiograms ruled out coronary aneurysms, maintaining a normal ventricular function. The patient was discharged from hospital on day 17. After a month of follow up, the patient remained asymptomatic.

## 3. Discussion

We report the first case of PIMS-TS, according to the definition proposed by the RCPCH, treated with Tocilizumab in Chile.

Several clusters of children from the UK and USA with this inflammatory syndrome were recently published [7,8,9,10]. The typical findings were, as in our patient, an acute febrile illness with gastrointestinal involvement, evidence of single or multiorgan dysfunction, elevated inflammatory parameters (C reactive protein, ferritin, fibrinogen, lactate dehydrogenase, D-dimer and leukocytosis), hypoalbuminemia, hypertriglyceridemia, and altered pro-inflammatory cytokines profile. These findings were different from those found in COVID-19 in children, which is usually asymptomatic or with mild respiratory symptoms.

In all reported cases [7,8,9,10], the diagnosis was established after the exclusion of common microbial causes. SARS-CoV-2 PCR or serological tests were positive in some patients. In those who were negative, the diagnosis was justified by close contact with a positive case for SARS-CoV-2 [7,8,9,10]. In our patient, the SARS-CoV-2 PCR was positive and at that time, there was no possibility of performing serology in our center.

The physiopathology of this syndrome remains unclear. In adults with severe COVID-19 a late or hyperinflammatory phase has been described and it is associated with a massive pro-inflammatory response, also known as a cytokine storm, characterized by high levels of inflammatory cytokines such as IL-6, IL-1b, or IL-10 [13,14]. This cytokine storm leads to an immune response uncontrolled and macrophage activation syndrome that produces Acute Respiratory Distress Syndrome (ARDS) and multiorgan dysfunction [15]. The most recent clinical experiences from China suggested that IL-6 is one of the most important cytokines involved in SARS-CoV-2′s cytokine storm [16,17]. High IL-6 levels have been correlated to a worse prognosis [18]. 

In children, many authors suggest that PIMS-TS is a delayed immune phenomenon associated with inflammation (late phase or hyperinflammatory) after symptomatic or asymptomatic SARS-CoV-2 infection [19], but this is unknown. An aberrant cellular or humoral adaptive immune response is proposed to be involved, as long as PIMS-TS occurs when PCR is negative, and antibodies against SARS-CoV-2 are positive [5,6,7,11,12]. Another possible hypothesis derives from coronaviruses’ well-known ability to block type I and type III interferon responses, producing a delayed cytokine storm response that causes a slower virus clearance and an increased inflammation [12]. 

PIMS-TS lacks established treatment and follow-up protocols. The cornerstone of the management of patients with severe SARS-CoV-2 is intensive care support. Plasma infusion from convalescent patients and antivirals have been proposed as adjuvant treatments, yet restricted to clinical trials. Multiple therapies have been proposed to curb the inflammatory cascade, including IVIG, corticosteroids, and biologics therapies such as anti-TNF therapy, Infliximab, IL-1 blockade (Anakinra), inhibitor IL-6 receptor (Tocilizumab) and JAK inhibition (e.g., Baricitinib). 

**Figure 2:**
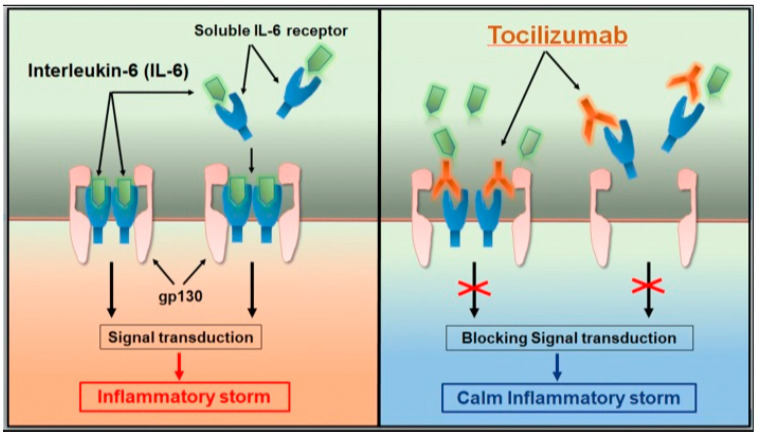
Tocilizumab calms the inflammatory storm through blocking IL-6 receptors [18].

As previously mentioned, IL-6 plays an essential role in the cytokine storm. Tocilizumab is a recombinant humanized monoclonal antibody against the human IL-6 receptor, which specifically binds soluble and membrane receptors, inhibiting their signal transduction [16,17,18] (Figure 2). Case reports using Tocilizumab in SARS-COV-2 infection have been published in adults [20,21], showing favorable outcomes in patients with extensive bilateral lung lesions opacity or in critical patients, especially when IL-6 is increased. Multiple trials are ongoing to study the safety and efficacy of this drug in COVID-19 [22]. The use of Tocilizumab in children is rarely reported. The most extensive pediatric experience to date described its use in 12 children in a case series of 33 patients with PIMS-TS [23]. It is recommended to initiate Tocilizumab with a single dose of 8 mg/kg in children over 30 kg, and 12 mg/kg when they are under this weight. In the case of inadequate response, an additional application of a similar dose can be made after 12 h [16]. Due to the administration of Tocilizumab, IL-6 levels in serum will temporarily increase within the next few days because the monoclonal antibody has blocked its receptors [20,22]. For all these reasons, together with the unavailability of other immunomodulatory drugs in Chile (e.g., Anakinra, Baricitinib), we decided to use Tocilizumab in our patient.

Although the concomitant use of corticosteroids indeed makes it difficult to determine whether the improvement in our patient was due to its use or because of Tocilizumab, we believe that the latter was decisive in clinical improvement due to the central role of IL-6 in the immune response.

The detection of increased biochemical inflammation markers (ferritin, D-dimer, triglycerides, LDH, and IL-6) and a rapid multiorgan worsening (including the development of ARDS) are critical to decide the timing of initiation of immunomodulatory therapy.

## 4. Conclusions

PIMS-TS is a new disease developed in a small percentage of patients, so a high degree of suspicion is necessary to establish the diagnosis. Supportive care is of paramount importance. The use of Tocilizumab to control the inflammatory response is likely to be beneficial, especially if IL-6 levels are elevated, but the best immunotherapeutic agent has not yet been established. Randomized clinical studies should be run to determine the best treatment.
